# An FDA-Drug Library Screen for Compounds with Bioactivities against Meticillin-Resistant *Staphylococcus aureus* (MRSA)

**DOI:** 10.3390/antibiotics4040424

**Published:** 2015-10-09

**Authors:** Qiu Ying Lau, Yoke Yan Fion Tan, Vanessa Chai Yin Goh, David Jing Qin Lee, Fui Mee Ng, Esther H. Q. Ong, Jeffrey Hill, Cheng San Brian Chia

**Affiliations:** Experimental Therapeutics Centre, Agency for Science, Technology and Research (ASTAR), 31 Biopolis Way, 2 Nanos #03-01, Singapore 138669, Singapore; E-Mails: qylau@etc.a-star.edu.sg (Q.Y.L.); xxnoif@gmail.com (Y.Y.F.T.); vanessa6382@hotmail.com (V.C.Y.G.); davrockyness@gmail.com (D.J.Q.L.); fmng@etc.a-star.edu.sg (F.M.N.); eong@etc.a-star.edu.sg (E.H.Q.O.); jhill@etc.a-star.edu.sg (J.H.)

**Keywords:** drug library screen, drug repurposing, drug repositioning, MRSA

## Abstract

The lack of new antibacterial drugs entering the market and their misuse have resulted in the emergence of drug-resistant bacteria, posing a major health crisis worldwide. In particular, meticillin-resistant *Staphylococcus aureus* (MRSA), a pathogen responsible for numerous human infections, has become endemic in hospitals worldwide. Drug repurposing, the finding of new therapeutic indications for approved drugs, is deemed a plausible solution to accelerate drug discovery and development in this area. Towards this end, we screened 1163 drugs approved by the Food and Drug Administration (FDA) for bioactivities against MRSA in a 10 μM single-point assay. After excluding known antibiotics and antiseptics, six compounds were identified and their MICs were determined against a panel of clinical MRSA strains. A toxicity assay using human keratinocytes was also conducted to gauge their potential for repurposing as topical agents for treating MRSA skin infections.

## 1. Introduction

Due to the overuse, misuse and lack of new antibiotics approved for sale in the past decade, the emergence of drug-resistant bacteria is now a universal threat to human health [1,2,3,4]. One notorious culprit, meticillin-resistant *Staphylococcus aureus* (MRSA), is the leading cause of human infections [5,6,7,8,9] and is primarily responsible for acute bacterial skin and skin structure infections (ABSSSIs) [10,11]. MRSA is now endemic in most hospitals, creating a huge medical and financial burden worldwide [3,12,13,14]. The Centers for Disease Control and Prevention (CDC) estimated that there were over 80,000 MRSA infection cases in the USA in 2011, resulting in more than 11,000 fatalities and having MRSA flagged as a serious threat to human health in their 2013 antibiotic resistance threat report [15].

One reason for the low numbers of new antibacterial drugs reaching the market is the perceived low return of investment [4]. As a result of regulatory hurdles, high research costs and long drug development timelines, many large pharmaceutical companies have been abandoning this research field since the late 1990s [4,16,17,18,19]. To address this, drug repurposing or repositioning—the finding of new therapeutic indications for approved drugs—has been proposed as a possible solution [3,20,21,22]. As the safety, pharmacodynamic profiles and manufacturing processes of approved drugs have already been established, they can be rapidly made available for a new disease indication as the time-to-market and developmental costs are considerably reduced [23].

Based on this, we screened 1163 drugs approved by the Food and Drug Administration (FDA) for antibacterial activities on a prevalent nosocomial MRSA strain (USA100) [24] in a single-point 10 μM assay. After identifying the hit compounds, known antibiotics and antiseptics were excluded from the list. The remaining compounds, together with six FDA-approved antibiotics approved for treating MRSA infections, were subjected to a minimum inhibitory concentration (MIC) assay against a panel of clinical MRSA strains for comparison. A human cell toxicity assay was conducted to determine the compounds’ suitability for repurposing as antibacterial agents for treating MRSA infections.

## 2. Results and Discussion

**Figure 1:**
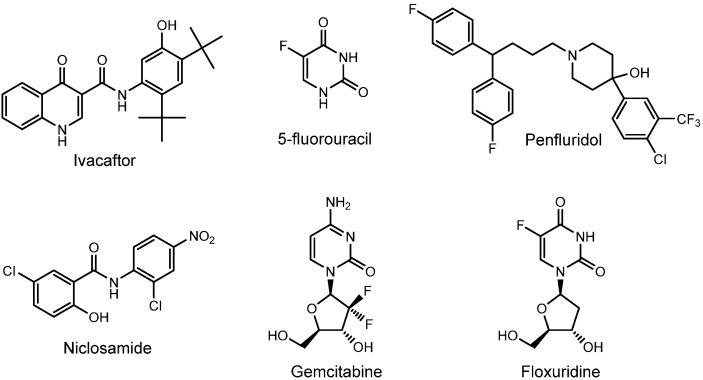
Drug structures with bioactivities against MRSA.

In the single-point 10 μM compound screen, 1163 FDA-approved drugs were tested for antibacterial activities using MRSA USA 100, the prevalent nosocomial MRSA strain found in US hospitals [24]. There were 43 compounds discovered to be either bacteriostatic or bactericidal (see supplementary data). After the exclusion of known antiseptics and antibiotics, six compounds were identified: Ivacaftor, 5-Fluorouracil, Penfluridol, Niclosamide, Gemcitabine hydrochloride and Floxuridine (Table 1, Figure 1). These compounds were then purchased from two vendors (see Experimental section) for MIC assays against a panel of clinical MRSA strains (USA 100, USA 300, USA 400 and VISA). Six antibiotics approved for treating MRSA infections were also included for comparison [25]. The six compounds, arranged in ascending order of antibacterial potencies, are discussed in detail hereafter. The most potent compound was subjected to a bacteriostatic/bactericidal determination assay followed by a MRSA time-kill assay to determine its potential for drug repurposing.

**Table 1:** Drug data, GI_50_s and MICs against various MRSA strains. Abbreviations: IV, intravenous; OR, oral; TO, topical.

Drugs Found in This Screen	Original Indication	Administration Route	Keratinocyte GI_50_ (μM)	MRSA MICs (μM)			
				USA 100	USA 300	USA 400	VISA
Ivacaftor	cystic fibrosis	OR	5.9 ± 0.5	6.25	6.25	6.25	6.25
5-fluorouracil	cancer	IV, TO	33.3 ± 2.6	6.25	3.13	3.13	6.25
Penfluridol	psychosis	OR	2.1 ± 0.6	3.13	3.13	3.13	3.13
Niclosamide	anthelmintic	OR	<0.2	0.78	0.39	0.78	0.39
Gemcitabine	cancer	IV	1.9 ± 0.2	0.10	0.05	0.10	0.05
Floxuridine	cancer	IV	>50	0.025	0.00313	0.0125	0.00625
FDA-approved MRSA drugs							
Clindamycin	MRSA infection	IV, OR, TO	>50	>100	0.20	0.20	>100
Doxycycline	MRSA infection	IV, OR	36.9 ± 3.7	0.39	0.78	1.56	12.5
Linezolid	MRSA infection	IV, OR	>100	6.25	6.25	6.25	3.13
Mupirocin	MRSA infection	TO	>50	0.78	0.39	0.39	>100
Tigecycline	MRSA infection	IV	20.6 ± 4.2	0.78	0.78	0.78	1.56
Vancomycin	MRSA infection	IV	>100	0.78	0.78	0.78	6.25

### 2.1. Ivacaftor

Quinolinone Ivacaftor (Kalydeco; Figure 1) is an oral cystic fibrosis transmembrane conductance regulator developed by Vertex Pharmaceuticals and was approved by the FDA in 2012 for the treatment of cystic fibrosis in patients bearing a G551D mutation [26]. Due to its structural similarity to the quinolone antibiotics, Reznikov and co-workers recently demonstrated that Ivacaftor was also active against various strains of *Staphylococcus aureus* with MICs ranging from 8 to 32 μg·mL^−1^ (20.4 to 81.5 μM) [27]. In our MIC assays, Ivacaftor exhibited a consistent MIC of 6.25 μM against our MRSA panel, on par with Linezolid (Table 1). Although shown to have low oral toxicity (LD_50_ 2.28 moL·kg^−1^) in rats [28], our human skin epidermal keratinocyte viability assay results revealed Ivacaftor to be relatively toxic towards human skin keratinocytes (GI_50_ 5.9 ± 0.5 μM, Table 1), close to its MIC of 6.25 μM against MRSA. Hence, we believe this drug may be too toxic for repurposing as a topical antibacterial for MRSA infections.

### 2.2. 5-Flurouracil

Pyrimidine analog 5-Fluorouracil (Adrucil; Figure 1) is an antimetabolite developed by Roche in the 1950s for the treatment of various cancers [29]. Its mechanism of action is thought to involve thymidylate synthase inhibition, a key enzyme involved in DNA synthesis [30,31]. In our MIC assays, 5-Fluorouracil exhibited MICs of 3.13 to 6.25 μM against our panel of MRSA, on par with Linezolid (Table 1). However, this intravenously administered drug has an extremely short half-life (10 to 16 min), is highly toxic (mouse intravenous LD_50_ 81 mg·kg^−1^) and causes a number of adverse side effects like cardiotoxicity and myelosuppression [31], thereby making it unsuitable, in our opinion, as an antibacterial drug for systemic and bacteremia treatment. In our human skin epidermal keratinocyte viability assay, 5-Fluorouracil displayed a GI_50_ of 33.3 ± 2.6 μM (Table 1), five-fold higher than its MIC against MRSA (6.25 μM). If this drug were to be repurposed as a topical antibacterial, a strict dosing regimen would have to be enforced. In 2000, a 0.5% topical cream (Carac) was approved for treating certain skin cancers and we believe similar creams can potentially be formulated for treating MRSA skin infections with a strict dosing regimen.

### 2.3. Penfluridol

Diphenylbutylpiperidine Penfluridol (Flupidol, Semap and Micefal; Figure 1) is an oral antipsychotic dopamine receptor inhibitor developed by Janssen in 1968 for the treatment of psychosis and chronic schizophrenia [32]. In our MIC assays, Penfluridol exhibited a consistent MIC of 3.13 μM against our MRSA panel, two-fold more potent than Linezolid (Table 1). However, due to its adverse side effects like epilepsy, akathisia, dyskinesia and pseudo-Parkinsonism, we believe Penfluridol is unsuitable for repurposing as an oral or intravenous antibacterial. In our human skin epidermal keratinocyte viability assay, Penfluridol was surprisingly toxic with a GI_50_ of 2.1 ± 0.6 μM (Table 1), almost on par to its MIC against MRSA (3.13 μM), suggesting that it may be too toxic to be redeveloped as a topical antibacterial agent.

### 2.4. Niclosamide

The salicyanilide Niclosamide (Niclocide; Figure 1) is an oral anthelmintic developed by Bayer and approved in 1961 for treating tapeworm infections in the gut. Its anthelmintic mechanism is suspected to involve the disruption of oxidative phosphorylation in tapeworms [33]. A very recent drug screening report involving MRSA-infected *C. elegans* also identified Niclosamide from an FDA drug library [34]. In our MIC assay, Niclosamide displayed potent MICs between 0.39 to 0.78 μM against our MRSA panel, up to two-fold more potent than the gold-standard MRSA antibiotic Vancomycin (Table 1) [5,35]. When taken orally, Niclosamide is minimally absorbed into the bloodstream from the gastrointestinal tract and has an oral LD_50_ of >1000 mg·kg^−1^ [36], suggesting it can be used for treating gastrointestinal tract infections caused by MRSA [37,38]. However, our human skin epidermal keratinocyte viability assay results revealed Niclosamide to be highly cytotoxic with a GI_50_ of <0.2 μM (Table 1), ruling out the possibility of it being repurposed as a topical antibacterial. Indeed, the US Environmental Protection Agency classified Niclosamide as a skin irritant [39] and the drug has been shown to be cytotoxic towards a range of human carcinoma cell lines at sub-micromolar concentrations via an unknown mechanism of action [40].

### 2.5. Gemcitabine

Nucleoside analog Gemcitabine (Gemzar; Figure 1) is an intravenously administered antimetabolite ribonucleotide reductase inhibitor developed by Eli Lilly and Company for the treatment of various cancers and was approved by the FDA in 1996 [41]. Gemcitabine was found to be very potent towards our MRSA panel with MICs ranging from 0.05 to 0.10 μM (0.01 to 0.02 μg·mL^−1^, Table 1). However, when administered intravenously, its short half-life (42 to 94 min), high toxicity (rat intravenous LD_50_ 236 mg·kg^−1^) and adverse side effects such as myelosuppression are likely to count against it as an antibacterial drug candidate for systemic infections and bacteremia [42]. However, a topical formulation developed for the treatment of endometrial, cervical and ovarian epithelial cancers progressed to phase 2 clinical trials in 2008 (NCT00610740) and, coupled with its high potency against MRSA, consideration should be given to this drug to be repurposed as a topical antibacterial. In our human skin epidermal keratinocyte viability assay, Gemcitabine displayed a GI_50_ of 1.9 ± 0.2 μM (Table 1), an approximate 40-fold difference compared to its MIC against MRSA USA300 (MIC 0.05 μM), suggesting its potential for repurposing as a topical antibacterial for MRSA ABSSSIs with a proper dosing regimen and treatment period.

### 2.6. Floxuridine

**Figure 2:**
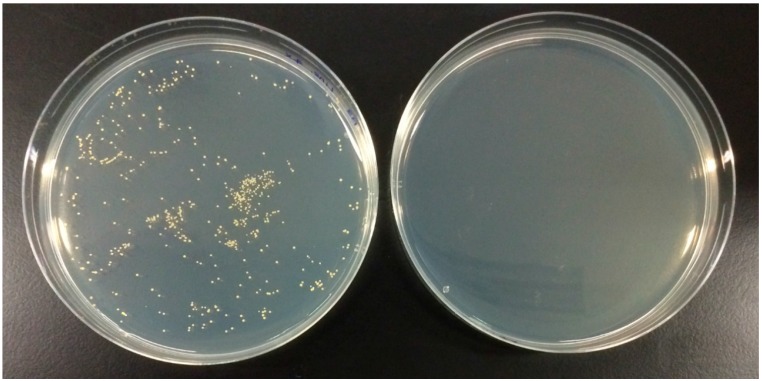
Bactericidal/static determination assay; left plate: Linezolid; right plate: Floxuridine.

**Figure 3:**
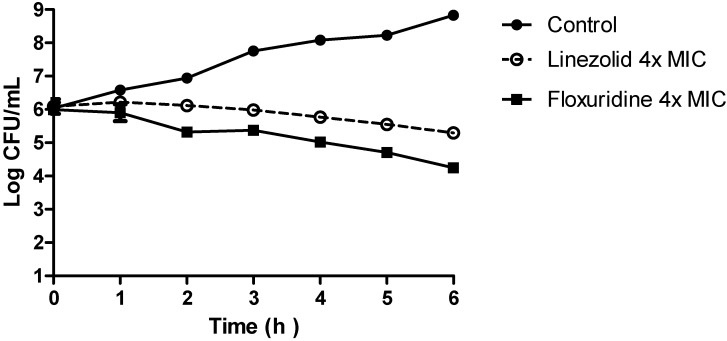
Time-kill assay of Linezolid and Floxuridine at 4× MICs using MRSA USA 300.

Nucleoside analog Floxuridine (FUDR; Figure 1) is an analog of 5-Fluorouracil developed by Roche for the treatment of various cancers. It is an intravenously administered antimetabolite thymidylate synthase inhibitor approved by the FDA in 1970 [43,44]. Of the six compounds identified in our screen, Floxuridine exhibited the highest potency in our MRSA panel with MICs between 0.00313 to 0.025 μM (0.00077 to 0.00616 μg·mL^−1^; Table 1). When administered intravenously, its adverse side effects including myelosuppression may count against it as an antibacterial drug candidate for systemic infections and bacteremia. However, due to its extremely high bioactivity against MRSA and its relatively low toxicity (rat intraperitoneal LD_50_ 1600 mg·kg^−1^) [45], we believe that Floxuridine can potentially be re-formulated as a topical agent and repurposed for treating ABSSSIs caused by MRSA. In our human skin epidermal keratinocyte viability assay, Floxuridine was found to be non-cytotoxic (GI_50_ of >100 μM, Table 1), making it the most promising compound amongst the six for repurposing as a topical antibacterial. A bacteriostatic/bactericidal determination assay using MRSA USA300 was subsequently conducted with a comparator drug, Linezolid. Antibiotics with a bactericidal mechanism of action are preferred because the bacteria, once killed, will not have the opportunity to multiply and develop resistance. As shown in Figure 2, Floxuridine was found to be bactericidal while Linezolid was bacteriostatic, in agreement with the literature [46]. A bacteria time-kill assay using MRSA USA300 showed Floxuridine caused an approximate 2-log reduction in bacteria count within 6 h at 4× MIC (Figure 3). Linezolid, in comparison, caused less than a log reduction in 6 h at 4× MIC, in agreement with the literature [47].

## 3. Experimental Section

### 3.1. Compound Library, Test Compounds and Bacteria

The 1163 FDA-approved drugs were purchased as a library set from Selleckchem (catalog #L1300). For MIC assays, Ivacaftor, Mupirocin and Tigecycline were purchased from Santa Cruz Biotechnology (Dallas, TX, USA). All other test compounds were purchased from Sigma-Aldrich (St. Louis, MO, USA) and were used without further purification. MRSA strains USA 100 (ATCCBAA-1681), USA 300 (ATCC-BAA-1680), USA400 (ATCC-BAA-1707) and VISA (ATCC-700699) were purchased from ATCC (Manassas, VA, USA).

### 3.2. Single-Point Bacteria Growth Inhibition Assay

MRSA USA100 was grown from frozen glycerol stock on Mueller Hinton 2 (MH2) agar overnight at 37 °C. Five colonies were selected from the agar plate and grown in cation-adjusted MH2 broth (BD Biosciences catalog #212322, Franklin Lakes, NJ, USA) in a shaker incubator at 37 °C, 220 RPM. Cells were grown to 1–2 × 10^8^ CFU/mL and diluted 1:100 in MH2 broth. An amount of 50 μL of test compounds pre-dissolved in MH2 broth at 20 μM concentrations were added in duplicates into each well in a 96-well plate. A total of 50 μL of bacteria inoculums containing ~1 × 10^6^ CFU/mL of bacteria in MH2 broth was introduced into each well containing the test compounds. The final DMSO concentration was kept at 2.5% in each well. After an overnight incubation at 35 °C, optical density (OD_600_) of each well was determined using the Microplate Spectrophotometer (Molecular Devices Spectra Max Plus, Sunnyvale, CA, USA).

### 3.3. Minimum Inhibitory Concentration (MIC) Assay

The MICs of antibiotics and peptides were determined using the microdilution method from the Clinical and Laboratory Standards Institute (CLSI) guidelines [48]. Briefly, bacteria were grown fresh from frozen stock in MH2 agar at 37 °C. After an overnight incubation, five MRSA colonies were selected to grow in cation-adjusted MH2 broth (BD Biosciences catalog #212322) in a shaker incubator at 37 °C, 220 RPM. Cells were grown to an optical density (OD_600_) of 0.15–0.16 using a Microplate Spectrophotometer (Molecular Devices Spectra Max Plus), which corresponds to 1–2 × 10^8^ CFU/mL. Test compounds were constituted into 4 mM DMSO stock solutions and then subjected to two-fold serial dilution in a 96-well plate with concentrations ranging from 100 to 0.2 μM in duplicates. The amount of 50 μL of microbial culture containing ~1–2 × 10^6^ CFU/mL of microbes in the respective broths was introduced into each well containing 50 μL of compound. After an overnight incubation at 35 °C, OD_600_ measurements were conducted using the Microplate Spectrophotometer. The MIC was defined as the lowest drug concentration (μM) required to inhibit microbial growth.

### 3.4. Human Epidermal Keratinocyte Viability (GI_50_) Assay

Adult normal human epidermal keratinocytes (NHEK) were purchased from Lonza (catalog #00192627, Basel, Switzerland) and cultured in keratinocyte growth medium (Lonza KGM-Gold Bullet Kit) containing growth supplements (Lonza Reagent Pack) at 5% CO_2_ (37 °C). The cell viability assay was performed using the CellTiter-Glo Luminescent Cell Viability Assay protocol from Promega (www.promega.com, Fitchburg, WI, USA). Briefly, NHEK were treated with test compounds in keratinocyte growth medium in 96-well plates (Thermo Scientific Nunclon Delta, Waltham, MA, USA). Treated cells were incubated for 5 days at 5% CO_2_ (37 °C). Promega CellTiter-Glo Reagent was added and plates were shaken on an orbital shaker for 2 h. Next, well contents (100 μL) from each well were transferred into opaque 96-well plates (PerkinElmer OptiPlate-96) and luminescence measured by a microplate reader (Tecan Safire2).

### 3.5. Bactericidal/Static Determination Assay

After the MICs of test compounds were determined using the microdilution method described in Section 2.2, the entire well contents (100 μL) corresponding to 4× the MIC of the antibiotic or peptide were transferred and spread on fresh sterile MH2 agar plates. The plates were incubated overnight at 37 °C and the number of colony forming units (CFU) was determined the next day. A compound was classified as bactericidal when ≥99.9% cell death was observed.

### 3.6. Time-Kill Assay

Bacteria cells were grown to 1 × 10^8^ CFU/mL in MH2 broth and then diluted 200-fold into 5 mL aliquots to a density of 1 × 10^6^ CFU/mL in a flask. Test compounds pre-dissolved in DMSO corresponding to 4× their MICs were then added into each flask with a final DMSO concentration of 2.5% *v*/*v*. The negative control was 2.5% DMSO. The flasks were incubated in a shaking incubator (220 RPM) at 37 °C. Samples (100 μL) were taken hourly from the flasks between *t* = 0 and *t* = 6 h. The samples were serially diluted in MH2 broth before plating on sterile MH2 agar and incubated overnight at 37 °C. A bacteria count for each plate was conducted the next day. A graph of Log CFU/mL was plotted against time to obtain the time-kill graph.

## 4. Conclusions

A total of 1163 FDA-approved drugs were screened for bioactivity against MRSA USA 100. Six drugs were identified with repurposing potential as antibacterial drugs for treating MRSA infections. After taking into account their MICs, known side effects, cellular toxicity, bactericidal mechanism of action and rapid MRSA killing ability, Floxuridine stood out as the most plausible candidate for repurposing as a topical agent for the treatment of MRSA ABSSSIs and thus warrants further investigations.

## Supplementary Files

Supplementary File 1
