# Supplementary material for: An FDA-Drug Library Screen for Compounds with Bioactivities against Meticillin-Resistant Staphylococcus aureus (MRSA)
Source: Antibiotics (Basel). 2015 Oct 9;4(4):424–34. doi: 10.3390/antibiotics4040424 (PMC4790305; doi:10.3390/antibiotics4040424)
Supplement: Supplementary File 1 [file antibiotics-04-00424-s001.pdf]

Supplementary Data

Drugs picked up from the single-point assay:

#	Plate#	Well	Drug
1	1	4c	Ivacaftor
2	1	4e	Gemcitabine HCl
3	1	7b	Fluorouracil
4	2	2f	Floxuridine
6	2	6g	Cefoselis sulfate
7	2	7a	Doripenem hydrate
5	2	9e	Teicoplanin
8	2	9h	Tigecycline
9	2	10d	Linezolid
10	3	2c	Moxifloxacin HCl
11	4	6b	Gemcitabine
12	4	8h	Rifabutin
13	4	10c	Rifapentine
14	4	11d	Oxytetracycline
15	4	10g	Rifampicin/Rifampin
16	5	1e	Rifaximin
17	5	1f	Bacitracin zinc
18	6	10c	Oxytetracycline dihydrate
19	7	7d	Sitafloxacin hydrate
20	7	7f	Tebipenem pivoxil
21	8	7g	Methacycline HCl
22	9	1d	Streptomycin sulfate
23	9	1f	Tetracycline HCl
24	9	1g	Vancomycin HCl
25	9	8f	Niclosamide
26	9	10h	Besifloxacin HCl
27	10	2d	Nadifloxacin
28	10	3e	Trimethoprim
29	10	10a	Doxycycline HCl
30	10	10b	Clinafloxacin
31	11	4d	Retapamulin
32	11	6e	Pyrithione zinc
33	11	10a	Clofazimine
34	12	1g	Penfluridol
35	12	2h	Benzethonium chloride
36	12	3h	Cetylpyridinium chloride
37	12	5f	Domiphen bromide
38	12	6d	Chlorquinaldol
39	12	8h	Valnemulin HCl
40	12	11e	Cetrimonium bromide
41	13	1c	Alexidine HCl
42	13	4e	Meclocyline sulfosalicylate
43	13	7e	Thonzonium bromide
